# Enhancement of astaxanthin accumulation via energy reassignment by removing the flagella of *Haematococcus pluvialis*

**DOI:** 10.1186/s40643-024-00789-x

**Published:** 2024-08-02

**Authors:** Yuyong Hou, Zhile Guo, Zhiyong Liu, Suihao Yan, Meijie Cui, Fangjian Chen, Weijie Wang, Longjiang Yu, Lei Zhao

**Affiliations:** 1 College of Life Science, North China University of Science and Technology, Tangshan, China; 2 Photosynthesis Research Center, Key Laboratory of Photobiology, Institute of Botany, Chinese Academy of Sciences, Beijing, China; 3 Key Laboratory of Engineering Biology for Low-carbon Manufacturing, Tianjin Institute of Industrial Biotechnology, Chinese Academy of Sciences, Tianjin, China; 4 National Center of Technology Innovation for Synthetic Biology, Tianjin, China; 5 University of Chinese Academy of Sciences, Beijing, China

**Keywords:** *Haematococcus pluvialis*, Astaxanthin, Flagella, Energy flow, pH-shock

## Abstract

Astaxanthin biosynthesis in *Haematococcus pluvialis* is driven by energy. However, the effect of the flagella-mediated energy-consuming movement process on astaxanthin accumulation has not been well studied. In this study, the profiles of astaxanthin and NADPH contents in combination with the photosynthetic parameters with or without flagella enabled by pH shock were characterized. The results demonstrated that there was no significant alteration in cell morphology, with the exception of the loss of flagella observed in the pH shock treatment group. In contrast, the astaxanthin content in the flagella removal groups was 62.9%, 62.8% and 91.1% higher than that of the control at 4, 8 and 12 h, respectively. Simultaneously, the increased Y(II) and decreased Y(NO) suggest that cells lacking the flagellar movement process may allocate more energy towards astaxanthin biosynthesis. This finding was verified by NADPH analysis, which revealed higher levels in flagella removal cells. These results provide preliminary insights into the underlying mechanism of astaxanthin accumulation enabled by energy reassignment in movement-lacking cells.

## Graphical abstract

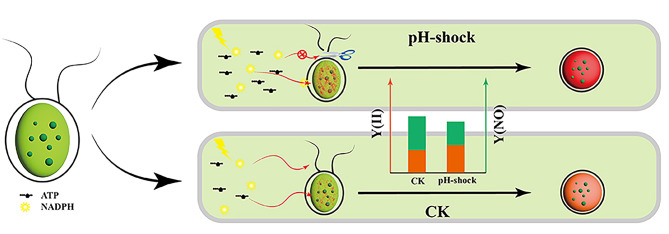

## Introduction

Natural astaxanthin, as a xanthophyll carotenoid, is one of the most powerful compounds with remarkable antioxidant activity and has been widely used in various fields, including medicine and food (Mahad et al., 2023). The chlorophyte microalgae *Haematococcus pluvialis* is established as the richest source of natural astaxanthin due to its ability to accumulate a large amount of astaxanthin up to 4% of the total dry cell weight. Extreme environmental stress-induced conditions that induce the generation of reactive oxygen species have been extensively studied and well developed to promote the accumulation of natural astaxanthin, including high light, nitrogen starvation, high temperature, salt stress, and high carbon resources (Zhao et al. 2020).

Reactive oxygen species (ROS) generated under environmental stress usually result in signal transduction and oxidative damage within the induction period. Multiple strategies have evolved to activate the antioxidative system, such as quenching ROS by biosynthesizing antioxidants such as astaxanthin and scavenging free radical substances to protect proteins and cells from damage (Li et al. 2021). Interestingly, the generation of an appropriate level of ROS facilitates the accumulation of carbohydrates by upregulating the activity of acetyl-CoA carboxylase (ACCase), and the enhanced carbon flux would be assigned to the production of astaxanthin (Li et al. 2023). Moreover, excessive carbon supply is another way to enhance carbon metabolism, thus facilitating astaxanthin production. It has been reported that 4.4 mM of acetate significantly enhanced the growth of *Haematococcus pluvialis*, but the cells were observed to contain a minimal amount of astaxanthin, resulting in a green color (Vidhyavathi et al. 2008). The addition of a higher concentration of acetate, up to 50 mM, resulted in the cells turning brown and the induction of carotenoid biosynthesis (Lv et al. 2022; Mansouri et al. 2022). Meanwhile, the expression of astaxanthin biosynthetic genes was determined to be upregulated in response to stress or high carbon supply, which provided an explanation for the observed biosynthesis of carotenoids (Vidhyavathi et al. 2008; Galarza et al. 2018; Mahadi et al. 2023).

Due to the high esterification level of astaxanthin molecules in *H. pluvialis*, both ATP and NADPH are required for the energy-intensive process of fatty acid biosynthesis, resulting in competition between the biosynthesis of astaxanthin and other physiological metabolic pathways (Harwood and Guschina 2009; Srinivasula et al. 2013; Xue et al. 2017; Zhao et al. 2020). Throughout the life cycle of *H. pluvialis*, a considerable quantity of carotenoids accumulated in conjunction with the cell type transformed from motile ones into red immotile spore cells by gradually losing the flagella (Li et al. 2019a, b; Feng et al. 2020; Zhang et al. 2023). These results prompted us to speculate that nonmotile cells without flagella may enhance astaxanthin biosynthesis by assigning more energy to the process than normal cells.

In analyzing cell motility, the majority of studies have concentrated on the well-established model organism *Chlamydomonas* (Sequeira et al. 2017; Boyd et al. 2018; Ahmad et al. 2022). With the protruding flagella from the cell surface, *Chlamydomonas* cells could swim towards or away from the light source enabled by the contraction and extension of microtubules, which highly relies on the efficient conversion of energy from ATP into mechanical work. Compared to extensive studies on *Chlamydomonas*, limited discussion has been reported on flagellar movement and its effect on astaxanthin accumulation in *H. pluvialis*. A previous study primarily tried to combine various conditions, including the addition of a high amount of carbon source and the removal of flagella to promote astaxanthin accumulation (Aflalo et al. 2007; Srinivasula et al. 2013; Han et al. 2020; Zhang et al., 2019). However, the underlying mechanism remains to be uncovered regarding the potential role of flagella removal in modulating astaxanthin biosynthesis.

In this study, rapid flagella removal in *Haematococcus* was carried out by pH shock and then used for further study after washing. The light energy absorbed by cells lacking flagella has not been negatively affected based on the results of photosynthetic activity measurements. The intracellular NADPH levels increased upon flagella removal, which would facilitate astaxanthin biosynthesis alongside the energy previously consumed by flagella-mediated movement. Thus, the present study provides new insight into the underlying mechanism of astaxanthin accumulation enabled by energy reassignment in motility-lacking cells in *H. pluvialis*. These findings will facilitate the improvement of astaxanthin accumulation, thereby enabling the development of more effective strategies to tackle future challenges related to natural products.

## Materials and methods

### Strain and culture conditions

The *Haematococcus pluvialis* (HA-3) strain used in this study was maintained at Tianjin Institute of Industrial Biotechnology, Chinese Academy of Sciences (Zhao et al. 2020). The algae seeds were cultivated to logarithmic phase with two flagella as previously described (Liu et al. 2020). The cells were concentrated by centrifugation at 5000 rpm for 5min and washed twice with 10 mM HEPES pH 7.4. Then the cells were transferred into a small beaker with stirring. The pH value of the medium was adjusted rapidly to 4.0 by the dropwise addition of 0.5 N acetic acid and incubated for 30 s, followed by neutralization to pH 7.0 through the dropwise addition of 0.5 M KOH (Luo et al. 2011; Han et al. 2020). The green vegetative cells were then collected by centrifugation at 3000 rpm for 5 min. Subsequently, the cell pellets were washed twice with sterile water and transferred to BG-11 medium with an initial cell density of 0.5 × 10^5^ cells mL^− 1^ under continuous light of 120 µmol photons m^− 2^s^− 1^ at 25 °C. HA-3 cells were harvested every 4 h for morphological observation, pigment analysis and fluorescence parameter analysis.

### Morphological observation and pigment determination

The cells harvested at the designated time points were used for the observation of morphological differences using a microscope with a camera (BX43F, COLYMPUS). For the determination of pigment contents, cells were harvested at 5000 rpm for 5 min. Then, the pellets were mixed with 1 mL of 5% KOH·30% CH_3_OH and incubated at 75 °C for 5 min to degrade chlorophyll. After centrifugation at 10,000 rpm for 5 min, the pellets were subjected to a 5-minute water bath at 75 °C with the addition of 1 mL of dimethylsulfoxide (DMSO) and 0.05 mL of acetic acid until the pellets became colorless. Astaxanthin content was determined in a spectrophotometer at a wavelength of 490 nm and calculated as described in a previous study (Zhao et al. 2020). To determine chlorophyll content, cell pellets were ground using liquid nitrogen. Then, 2 mL of methanol was added to the samples, and the mixture was incubated at 4 °C for 24 h. The OD663 and OD645 values were measured using spectrophotometry (UV-1800PC, Shanghai Mapada Instrument., LTD) and then used for the calculation of chlorophyll contents according to the following formula (Ma et al. 2023), where V represents the volume of microlgae for pigment extraction.

Chl a (mg/L)=(12.7×OD663-2.69×OD645)×2/V;

Chl b (mg/L)=(22.9×OD645-4.86×OD663)×2/V;

Chl (mg/L) = Chl a + Chl b

### Measurement of chlorophyll fluorescence parameters and NADPH contents

An IMAGING-PAM fluorometer (Walz, Germany) was used to measure chlorophyll fluorescence parameters according to the manufacturer’s instructions. The maximum photochemical efficiency (Fv/Fm), actual photosynthetic efficiency of photosystem II (Y(II)), the effective quantum yield (Y(NO)) and the quantum yield of regulatory energy dissipation (Y(NPQ)) were determined. To quantify the NADPH content, cells were frozen in liquid nitrogen, ground at 4 °C, and then the NADPH content was measured by using an NADPH Assay Kit (S0179, Beyotime).

### Statistical analysis

For the statistical analysis, each value was evaluated using three biological replicates. Values are reported as the mean ± standard deviation. Data analysis was performed by one-way ANOVA using SPSS Statistics software. A significant difference was considered at the level of *p* < 0.05.

## Results and discussion

### Establishment of a pH shock method to remove flagella in *H. pluvialis*

**Fig. 1:**
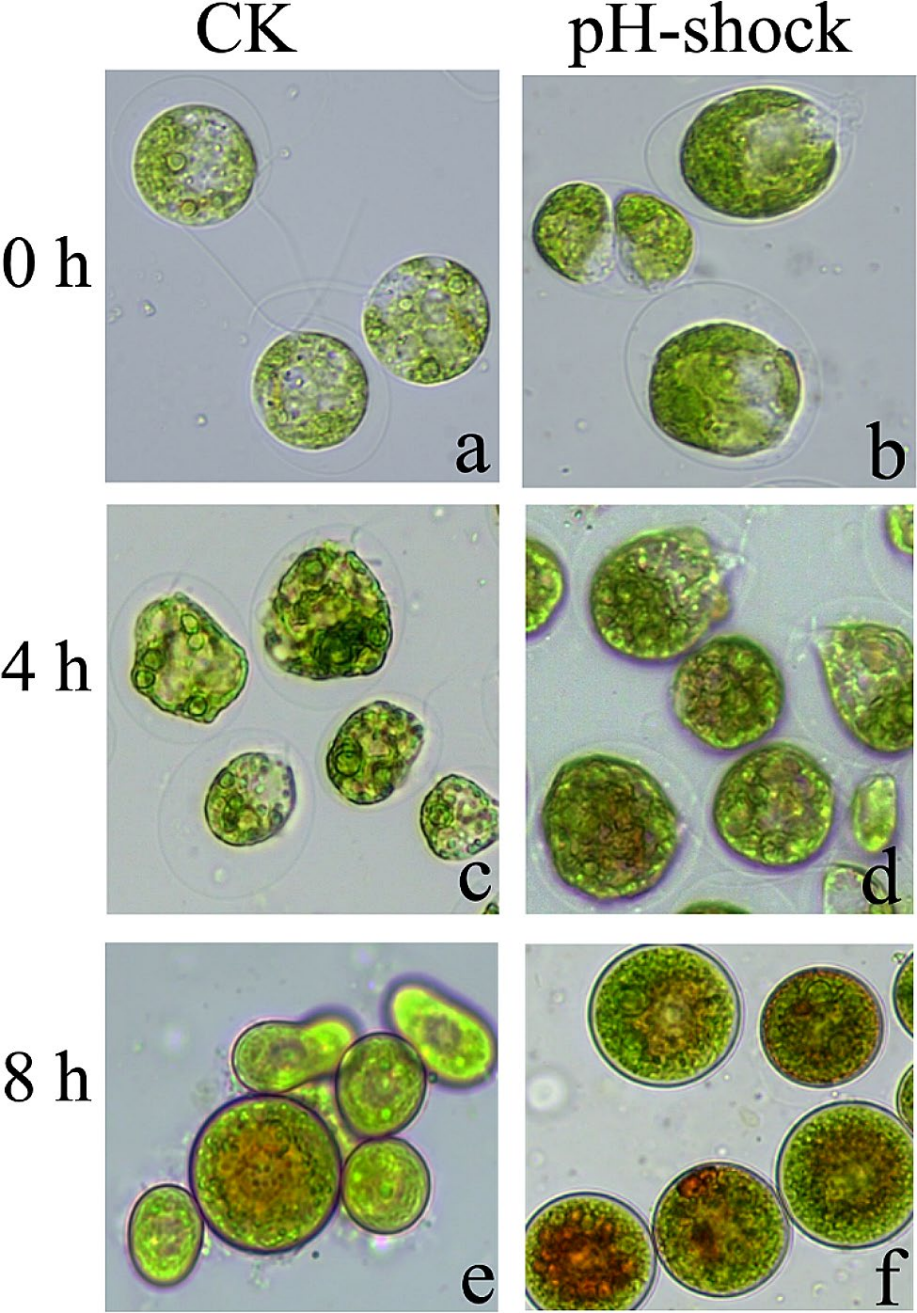
The morphological changes of control and flagella removal cells at three distinct time points (0, 4, and 8 h). Representative photos from each experiment are presented here

The pH shock method has been empolyed to study flagellar motility in *Chlamydomonas* (Tian et al. 2009). To clarify the possible effect of flagellar motility on astaxanthin biosynthesis in *H. pluvialis*, we implemented and repeated the pH shock protocol as reported in a previous study (Luo et al. 2011). The flagella of *H. pluvialis* were indeed removed after pH shock compared to those in the control group (Fig. 1). Both the extracellular matrix and cell type were observed with no significant difference between the control and treatment groups, as transparent extracellular matrix and green oval cells were detected in both groups. However, the extracellular matrix of the treated cells narrowed, and the cells were detected with a light brown color at 4 h after pH shock (Fig. 1). Then, the cells underwent a change in color, becoming darker brown at 8 h, and immotile spore cells were observed in the experimental group, indicating the activation of astaxanthin biosynthesis (Zhao et al. 2020). Based on the established pH shock method here, we analyzed the potential impact of flagellar motility on astaxanthin biosynthesis in *H. pluvialis*.

### Astaxanthin biosynthesis is enhanced by flagella removal

**Fig. 2:**
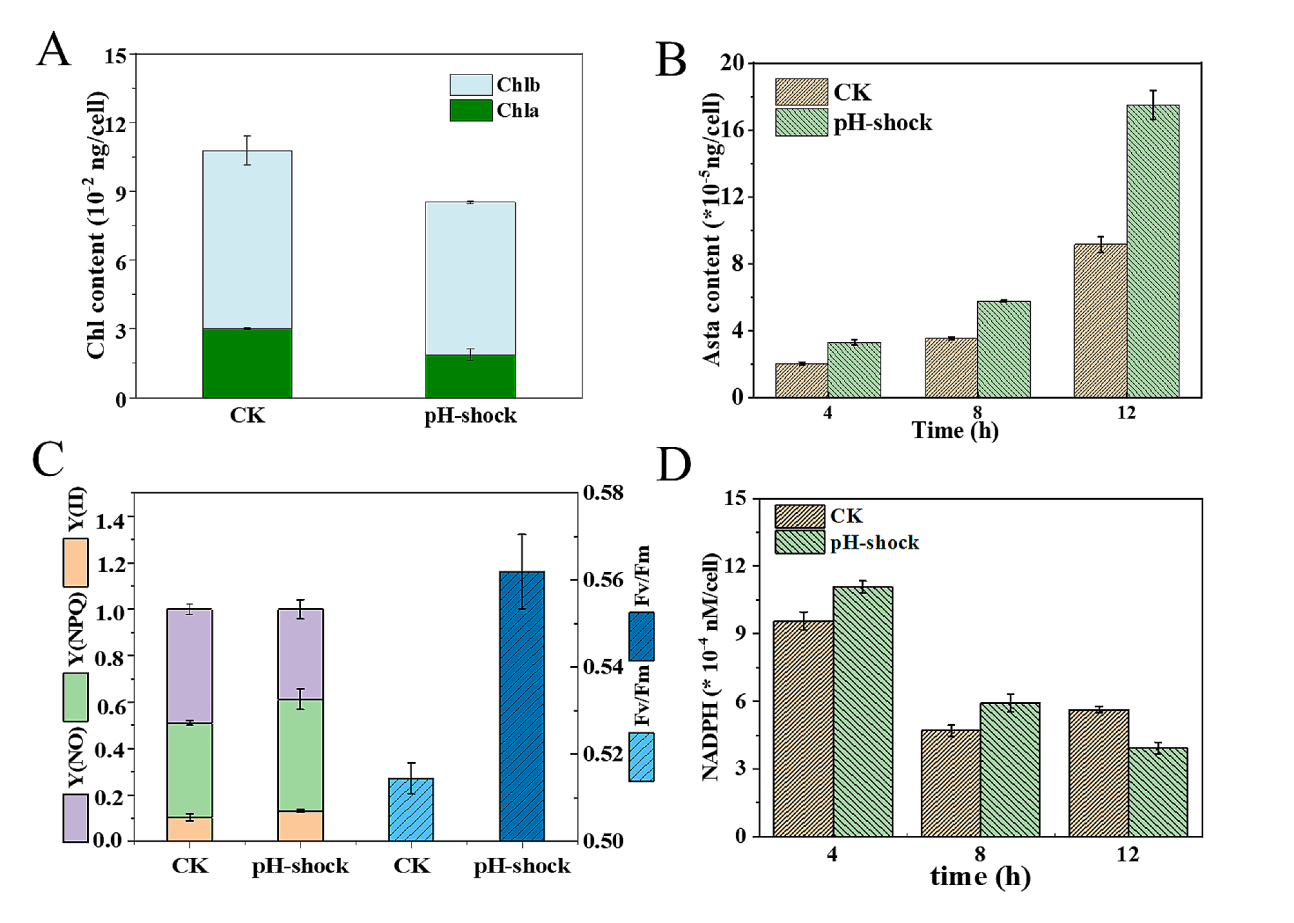
The effect of flagellar removal on chlorophyll contents (**A**), astaxanthin contents (**B**), chlorophyll fluorescence parameters (**C**) and NADP^+^/NADPH content (**D**) after different hour-treatments in *H. pluvialis*. Chlorophyll contents measurements were carried out after eight hour-treatment. Chl, chlorophyll; Asta, astaxanthin

To assay the possible effect of flagella removal on pigment biosynthesis, we measured chlorophyll and astaxanthin contents at the set time points. As shown in Fig. 2A, chlorophyll contents were decreased in the treatment group compared to those in the control group. These results indicated that either chlorophyll biosynthesis is inhibited or chlorophyll degradation is activated by pH shock. However, astaxanthin was significantly increased by 62.9%, 62.8% and 91.1% after flagella removal compared to the control samples at 4, 8 and 12 h, respectively (Fig. 2B). These results suggest that astaxanthin biosynthesis was activated and that the energy flux had been adjusted in favor of its biosynthesis by the treatment. As ATP is the energy resource for motility, flagella removal may benefit the energy requirements of the astaxanthin biosynthetic process (Ginger et al. 2008).

### NADPH may be induced as the key factor for astaxanthin biosynthesis by flagella removal

NADPH is an essential cofactor for both antioxidant defense and reductive biosynthesis (Yang et al. 2023), and its availability may be a key factor in regulating carbon flux to astaxanthin biosynthesis. Thus, modulation of NADPH homeostasis might be an effective strategy to enhance natural products.

Since parts of the astaxanthin biosynthetic pathway are located in chloroplasts, NADPH could be generated through photosynthesis. To further investigate the impact of flagella removal on NADPH production, we measured both chlorophyll fluorescence parameters and NADPH contents. As shown in Fig. 2C, the Fv/Fm and Y(II) values in the experimental group increased by 9.2% and 28.3% compared to those in the control group at 8 h, respectively, indicating improvement in both maximum and actual photochemical efficiency. The pH-shocked treatment seems to have the potential to moderately impact cellular photosynthetic activity, leading to an increase in NADPH generation. Considering the elevated levels of astaxanthin observed in the treatment group and the fact that astaxanthin’s primary structure is carbon-based, it can be speculated that more carbon flux and NADPH may have been assigned to the biosynthesis pathway of astaxanthin following flagella removal compared to the control group. Additionally, a higher Y(NPQ) indicated that the cells in the pH-shock group possessed a superior ability to maintain photoprotective ability compared to those in the control (Approbato et al. 2023; Gebara et al. 2023). Similarly, the experimental group exhibited a lower Y(NO) value, which suggested that flagella removal may favor the cells to protect themselves. Therefore, carbon flux and energy assignment could have been adjusted to favor astaxanthin biosynthesis by flagella removal, which is the main energy-consuming process under normal conditions.

To validate this hypothesis, we further determined the amount of available NADPH in cells. The intracellular NADPH of the flagella removal group was 15.6% and 26.1% higher than those in the control at 4 h and 8 h, respectively (Fig. 2D). Simultaneously, the higher NADPH could result in more ROS generation, which in turn prompted the super antioxidant biosynthesis to scavenge free radicals (Li et al. 2023; Yang et al. 2023). The yielding NAPDH could be constantly used for astaxanthin accumulation in *H. pluvialis* over time, thus resulting in a decreased level after 12 h. As a result, the astaxanthin contents in both groups showed increased profiles. In particular, the astaxanthin content of the pH shock group was 91.1% higher than those in control (Fig. 2B). Correspondingly, the total amount of NADPH was 30.3% lower in the pH shock group than in the control group at 12 h, suggesting its participation in astaxanthin accumulation. Therefore, removal of flagella could favor astaxanthin biosynthesis by generating more ATP and NADPH.

## Conclusion

To dissect the possible effect of cell motility and elucidate the mechanism of astaxanthin biosynthesis, we developed a flagella removal method for *H. pluvialis*. The removal of flagellar motility, which consumes both ATP and NADPH, allows for the reassignment of carbon and energy flux to the biosynthesis of natural products such as astaxanthin. The study here provides preliminary clues to the underlying mechanism of astaxanthin accumulation enabled by flagella removal. These findings will favor the improvement of astaxanthin accumulation by biotechnological efforts to tackle future challenges.

## Data Availability

All data generated or analyzed during this study are included in this published article and its additional files. The authors are willing to provide any additional data and materials related to this research that may be requested for research purposes.

## References

[CR1] Aflalo C, Meshulam Y, Zarka A, Boussiba S (2007) On the relative efficiency of two vs. one stage production of astaxanthin by the green alga *Haematococcus pluvialis*. Biotechnol Bioeng 98(1):300–305 17318905 10.1002/bit.21391

[CR2] Ahmad R, Bae AJ, Su YJ, Pozveh SG, Bodenschatz E, Pumir A, Gholami A (2022) Bio-hybrid micro-swimmers propelled by flagella isolated from *C. Reinhardtii*. Soft Matter 18(25):4767–4777 35703562 10.1039/D2SM00574C

[CR3] Approbato AU, Contin DR, Dias de Oliveira EA, Habermann E, Cela J, Pintó-Marijuan M, Munné-Bosch S, Martinez CA (2023) Adjustments in photosynthetic pigments, PS II photochemistry and photoprotection in a tropical C4 forage plant exposed to warming and elevated CO_2_. Plant Physiol Biochem 194:345–336 36463636 10.1016/j.plaphy.2022.11.033

[CR4] Boyd M, Rosenzweig F, Herron MD (2018) Analysis of motility in multicellular *Chlamydomonas reinhardtii* evolved under predation. PLoS ONE 13(1):e0192184 29381766 10.1371/journal.pone.0192184 PMC5790280

[CR5] Feng L, Zhang J, Fei Z, Wan M (2020) Astaxanthin accumulation difference between non-motile cells and akinetes of *Haematococcus pluvialis* was affected by pyruvate metabolism. Bioresour Bioprocess 7:5 10.1186/s40643-019-0293-1

[CR6] Galarza JI, Gimpe JA, Rojas V, Arredondo-Vega BO, Henríquez V (2018) Over-accumulation of astaxanthin in *Haematococcus pluvialis* through chloroplast genetic engineering. Algal Res 31:291–297 10.1016/j.algal.2018.02.024

[CR7] Gebara RC, Alho LOG, Mansano AS, Rocha GS, Melão MGG (2023) Single and combined effects of Zn and Al on photosystem II of the green microalgae *Raphidocelis subcapitata* assessed by pulse-amplitude modulated (PAM) fluorometry. Aquat Toxicol 254:106369 36502662 10.1016/j.aquatox.2022.106369

[CR8] Ginger ML, Portman N, McKean PG (2008) Swimming with protists: perception, motility and flagellum assembly. Nat Rev Microbiol 6(11):838–850 18923411 10.1038/nrmicro2009

[CR22] Gu W, Xie X, Gao S, Zhou W, Pan G, Wang G (2013) Comparison of different cells of Haematococcus pluvialis reveals an extensive acclimation mechanism during its aging process: from a perspective of photosynthesis. PLoS One 8(7):e67028 23922648 10.1371/journal.pone.0067028 PMC3724872

[CR9] Han SI, Chang SH, Lee C, Jeon MS, Heo YM, Kim S, Choi YE (2020) Astaxanthin biosynthesis promotion with pH shock in the green microalga, *Haematococcus lacustris*. Bioresour Technol 314:123725 32615445 10.1016/j.biortech.2020.123725

[CR10] Harwood JL, Guschina IA (2009) The versatility of algae and their lipid metabolism. Biochimie 91(6):679–668 19063932 10.1016/j.biochi.2008.11.004

[CR11] Li F, Cai M, Lin M, Huang X, Wang J, Ke H, Zheng X, Chen D, Wang C, Wu S, An Y (2019a) Differences between motile and nonmotile cells of *Haematococcus pluvialis* in the production of astaxanthin at different light intensities. Mar Drugs 17:39 30634492 10.3390/md17010039 PMC6356902

[CR12] Li F, Cai M, Lin M, Huang X, Wang J, Zheng X, Wu S, An Y (2019b) Accumulation of astaxanthin was improved by the nonmotile cells of *Haematococcus pluvialis*. BioMed Res Internat 8101762 10.1155/2019/8101762 PMC6379868 30868075

[CR13] Li Q, Zhao Y, Ding W, Han B, Geng S, Ning D, Ma T, Yu X (2021) Gamma-aminobutyric acid facilitates the simultaneous production of biomass, astaxanthin and lipids in *Haematococcus pluvialis* under salinity and high-light stress conditions. Bioresour Technol 320:12441 10.1016/j.biortech.2020.124418 33221643

[CR14] Li Q, Li L, Zhang Y, Gao H, Zhao Y, Yu X (2023) Chemical inducers regulate ROS signalling to stimulate astaxanthin production in *Haematococcus pluvialis* under environmental stresses: a review. Trends Food Sci Technol 136:181–193 10.1016/j.tifs.2023.04.014

[CR15] Liu Z, Hou Y, He C, Wang X, Chen S, Huang Z, Chen F (2020) Enhancement of linoleic acid content stimulates astaxanthin esterification in *Coelastrum* Sp. Bioresour Technol 300:122649 31896045 10.1016/j.biortech.2019.122649

[CR16] Luo M, Cao M, Kan Y, Li G, Snell W, Pan J (2011) The phosphorylation state of an Aurora-like kinase marks the length of growing Flagella in *Chlamydomonas*. Curr Biol 21(7):586–591 21458267 10.1016/j.cub.2011.02.046 PMC3075388

[CR17] Lv R, Liu K, Chen F, Xing H, Xu N, Sun X, Hu C (2022) Buffering culture solution significantly improves astaxanthin production efficiency of mixotrophic *Haematococcus pluvialis*. Bioresour Technol 354:127175 35452826 10.1016/j.biortech.2022.127175

[CR18] Ma X, Ren Y, Gao H, Wu X, Chen K, Khan R (2023) Proteomic, transcriptomic, biochemical, and physio-anatomical analyses provide insights into energy metabolism, light usage, and photosynthesis in cigar tobacco under different light intensities. Ind Crops Prod 198:116651 10.1016/j.indcrop.2023.116651

[CR19] Mahadi R, Kim S, Ilhamsyah DPA, Vahisan LPS, Narasimhan AL, Park GW, Lee SY, Oh YK (2023) Rapid accumulation of astaxanthin in *Haematococcus pluvialis* induced by mild hydrostatic pressure. Biotechnol Bioprocess Eng 28(2):345–351 10.1007/s12257-023-0017-4

[CR20] Mansouri H, Ebrahim Nezhad S, Kamyab H, Chelliapan S, Kirpichnikova I (2022) The effects of aeration and mixotrophy by acetate and pyruvate on the growth parameters in *Scenedesmus obliquus*. Biomass Convers Biorefinery 12(10):4611–4620 10.1007/s13399-022-02676-x

[CR21] Sequeira MP, Sinha S, Motiwalla MJ, Rao VG, D’Souza JS (2017) Defects in the ratio of the dynein isoform, DHC11 in the long-flagella mutants of *Chlamydomonas reinhardtii*. Biochem Biophys Res Commun 482(4):610–614 27865833 10.1016/j.bbrc.2016.11.081

[CR23] Tian P, Luo MN, Wang L, Guo Y, Li D, Li P, Snell WJ, Pan JM (2009) A microtubule depolymerizing kinesin functions during both flagellar disassembly and flagellar assembly in *Chlamydomonas*. PNAS 106(12):4713–4718 19264963 10.1073/pnas.0808671106 PMC2660737

[CR24] Vidhyavathi R, Venkatachalam L, Sarada R, Ravishankar GA (2008) Regulation of carotenoid biosynthetic genes expression and carotenoid accumulation in the green alga *Haematococcus pluvialis* under nutrient stress conditions. J Exp Bot 59(6):1409–1418 18343887 10.1093/jxb/ern048

[CR25] Xue J, Balamurugan S, Li DW, Liu YH, Zeng H, Wang L, Yang WD, Liu JS, Li HY (2017) Glucose-6-phosphate dehydrogenase as a target for highly efficient fatty acid biosynthesis in microalgae by enhancing NADPH supply. Metab Eng 41:212–221 28465173 10.1016/j.ymben.2017.04.008

[CR26] Yang J, Lee Y, Hwang CS (2023) The ubiquitin–proteasome system links NADPH metabolism to ferroptosis. Trends Cell Biol 33(12):1088–1103 37558595 10.1016/j.tcb.2023.07.003

[CR27] Zhang C, Zhang L, Liu J (2019) Exogenous sodium acetate enhances astaxanthin accumulation and photoprotection in *Haematococcus pluvialis* at the non-motile stage. J Appl Phycol 31:1001–1008 10.1007/s10811-018-1622-z

[CR28] Zhang L, Hu T, Yao S, Hu C, Xing H, Liu K, Sun X, Xu N (2023) Enhancement of astaxanthin production, recovery, and bio-accessibility in *Haematococcus pluvialis* through taurine-mediated inhibition of secondary cell wall formation under high light conditions. Bioresour Technol 389:129802 37783237 10.1016/j.biortech.2023.129802

[CR29] Zhao Y, Hou Y, Chai W, Liu Z, Wang X, He C, Hu Z, Chen S, Wang W, Chen F (2020) Transcriptome analysis of *Haematococcus pluvialis* of multiple defensive systems against nitrogen starvation. Enzyme Microb Technol 134:10948 10.1016/j.enzmictec.2019.109487 32044034

